# Dental Surgery

**Published:** 1868-02

**Authors:** B. T. Whitney


### Dental Surgery.

Editor Buffalo Medical and Surgical Journal:

There is an effort now being made, which is pushed forward with vigor, to bring before the present Legislature a bill, which we hope may become a law, to regulate the study and practice of dentistry, and for the formation and control of dental societies in this State. So far, dentistry has had no legal status, no less in regard to the qualification of those to be entrusted with the care and treatment of the teeth, or the diseases connected with them. The mass of people who may be most benefited by conservative dentistry, have no means of judging of qualification, except by sad experience in the loss of their natural teeth. A large mass of dentists have no knowledge of, or ability, to save the natural teeth, or to give them proper care—are uneducated in anything but the manual labor, as mechanics, in pulling them out and putting in artificial ones.

While we may not be able to weed out that class, or even to place restrictions upon any that are already in the business, this law will have the effect, so much desired, of demanding and receiving an established term of pupilage, as that of the medical student, an examination by a competent board of censors, who shall decide upon their qualification, and the result of this examination will be evidence to the community where he may locate, of his qualification. This will secure a better educated class of men, and a much higher standard of qualification of all who are hereafter to come into dental practice.

The bill, as framed, is much the same as the law pertaining to the medical profession—remodeled from it—but so changed as to meet the wants of the dental profession. The State and local societies are to be legalized on the same principles.

The following preamble and resolution were adopted by the New York State Medical Society, at its session just held in Albany:

*Whereas*, The Dental Profession of the State of New York, now numbering about two thousand practitioners, are about to petition the Legislature of the State for such legal enactments as will tend to regulate the practice of Dental Surgery, and to make some distinctions between the meritorious and skillful, and the ignorant pretender, and to give this profession a legal recognition, it is by this, the Medical Society of the State of New York.

*Resolved*, That this move on the part of the Dental Profession of this State, to procure such general laws for its protection, as now pertain to the medical profession, meets with our hearty approval, and that we hereby join in the prayer of their petition for this purpose.

The Erie County Medical Society and the Buffalo Medical Association passed similar resolutions at their January meetings. The Syracuse *Courier* speaking of Dental Surgery, says: "We are glad to see the medical profession as such, and in official as well as private capacities, recognizing and acknowledging the merits of this scientific art, not merely as an art, but as a collateral branch of the *healing* art, as a specialty of general surgery. Why this profession has not long since applied to our Legislature for some enactment to regulate its practice, and to protect the public against imposition from dental quackery, has to us been a wonder. But we see they have now gone about this work in earnest, and it is pleasant to see the *medical* profession promptly stepping forward to aid them to perfect this desirable object."

Yours, &c.,

B. T. Whitney.

